# Bridging Minds and Hearts by Exploring the Comorbidities Between Anxiety, Depression, and Takotsubo Cardiomyopathy: A Scoping Review

**DOI:** 10.3390/jcm15166242

**Published:** 2026-08-12

**Authors:** Michael Trung Nguyen, Malek Fajraoui, Alexandre Hudon

**Affiliations:** 1 Department of Psychiatry and Addictology, Faculty of Medicine, Université de Montréal, 2900 Bd Édouard-Montpetit, Montreal, QC H3T 1J4, Canada; michael.trung.nguyen@umontreal.ca (M.T.N.);; 2 Department of Psychiatry, Institut Universitaire en Santé Mentale de Montréal, 7401 Rue Hochelaga, Montreal, QC H1N 3M5, Canada; 3 Centre de Recherche de l’Institut Universitaire en Santé Mentale de Montréal, 7331 Rue Hochelaga, Montreal, QC H1N 3V2, Canada; 4 Department of Psychiatry, Institut National de Psychiatrie Légale Philippe-Pinel, 10905 Boul Henri-Bourassa E, Montreal, QC H1C 1H1, Canada; 5 Groupe Interdisciplinaire de Recherche sur la Cognition et le Raisonnement Professionnel, Université de Montréal, 2900 Bd Édouard-Montpetit, Montreal, QC H3T 1J4, Canada

**Keywords:** Takotsubo cardiomyopathy, Takotsubo syndrome, stress cardiomyopathy, anxiety disorders, depressive disorders, mental health, psychiatric comorbidity, brain-heart axis, psychocardiology, scoping review

## Abstract

**Background/Objectives:** Takotsubo cardiomyopathy (TTC), also known as “broken heart syndrome”, is an acute and reversible cardiac condition often triggered by emotional or physical stress. Increasing evidence suggests a strong comorbidity between TTC and psychiatric disorders, particularly anxiety and depression. However, the nature, prevalence, and implications of these associations remain incompletely understood. This scoping review aimed to systematically map the available evidence on the association between Takotsubo cardiomyopathy and anxiety and depressive disorders, characterize the prevalence and nature of these psychiatric comorbidities, evaluate the methods used for their assessment, and identify knowledge gaps to guide future research and clinical practice. **Methods**: A systematic search of MEDLINE (via PubMed), Embase, Web of Science, and Google Scholar was conducted for peer-reviewed human studies published up to December 2025 in English or French. Eligible studies reported on psychiatric comorbidities in TTC populations, including pre-existing disorders, psychiatric triggers, or psychological sequelae. A standardized extraction grid and the Joanna Briggs Institute appraisal tools were used for data collection and quality assessment. **Results:** Eighteen studies were included. Depression and anxiety were prevalent among TTC patients, frequently preceding or accompanying cardiac events. Emotional stressors such as bereavement or panic attacks were commonly cited as triggers. Despite this, only a minority of studies used validated psychometric tools, and longitudinal psychiatric follow-up was rare. Most studies were limited by small sample sizes, retrospective designs, and heterogeneity in diagnostic approaches. **Conclusions**: The findings reveal a consistent association between TTC and affective disorders, underscoring the role of the brain–heart axis. Integrating psychiatric screening and follow-up into TTC management may enhance outcomes. Future interdisciplinary research is needed to clarify causal pathways and inform prevention and treatment strategies.

## 1. Introduction

### 1.1. Takotsubo Cardiomyopathy

Takotsubo cardiomyopathy (TTC), also referred to as stress-induced cardiomyopathy, apical ballooning syndrome, or colloquially as “broken heart syndrome” is an acute, reversible cardiac condition that closely mimics the clinical presentation of an acute coronary syndrome (ACS), such as myocardial infarction, but occurs in the absence of significant obstructive coronary artery disease on angiography [1,2]. First described in Japan in the early 1990s, the syndrome was named “Takotsubo” after a traditional Japanese octopus trap, due to the characteristic ballooning of the left ventricular apex seen during systole on echocardiography and ventriculography [3]. Patients with TTC typically present with sudden onset chest pain, electrocardiographic changes (such as ST-segment elevation or T wave inversions), and elevated cardiac biomarkers like troponins, which further contribute to the initial diagnostic confusion with acute myocardial infarction [4,5].

Importantly, Takotsubo cardiomyopathy is precipitated in many cases by a sudden emotional or physical stressor (such as bereavement, interpersonal conflict, natural disasters, or severe medical illness), hence its designation as “stress-induced” [6,7,8]. Emotional triggers are more common than physical ones and often precede symptom onset by hours to days [9]. Although cardiac function in most TTC cases recovers spontaneously within days to weeks, the syndrome is not benign. It can lead to serious complications, including acute heart failure, ventricular arrhythmias, left ventricular thrombus formation, mitral regurgitation, and in some cases, cardiogenic shock or sudden cardiac death [10,11]. In-hospital mortality has been reported at rates comparable to those observed in acute coronary syndromes, particularly in patients with physical stressors or critical illness as triggers.

Epidemiologically, Takotsubo cardiomyopathy has a striking predilection for older women, particularly those postmenopausal, who constitute approximately 80–90% of all reported cases [2,12,13]. The underlying reasons for this demographic skew are not entirely understood but may relate to hormonal influences on vascular reactivity and stress responsiveness, including reduced estrogen-mediated cardioprotection. TTC is estimated to account for 1–2% of all cases initially suspected to be ACS and up to 6–10% of such cases in women presenting with ST-segment elevation and unobstructed coronary arteries [14]. With growing awareness and improved diagnostic tools such as cardiac MRI, the recognition and diagnosis of TTC have increased in recent years, though it remains underdiagnosed, especially in emergency settings [15].

From a pathophysiological standpoint, the precise mechanisms underlying Takotsubo cardiomyopathy remain incompletely understood [16]. The most widely accepted theory implicates a surge in catecholamines, such as epinephrine and norepinephrine, triggered by acute stress, leading to direct myocardial toxicity, coronary microvascular dysfunction, or transient left ventricular outflow tract obstruction [17]. These neurohormonal cascades are believed to result in myocardial stunning and the characteristic regional wall motion abnormalities observed in TTC [17]. Supporting this hypothesis are findings of markedly elevated plasma catecholamine levels in Takotsubo patients (often several times higher than in patients with myocardial infarction) [18]. However, other mechanisms, including neurogenic stunned myocardium, endothelial dysfunction, and genetic predisposition, have also been proposed. Current research continues to explore these multifactorial pathways, including how the brain–heart axis may mediate this unique syndrome through interactions between the central autonomic network and the cardiovascular system.

### 1.2. Mental Health Implications of Takotsubo Syndrome

The colloquial term “broken heart syndrome” for Takotsubo cardiomyopathy underscores the critical link between intense emotional states and cardiac events. A substantial body of evidence indicates that TTC has important comorbid associations with mental health conditions, particularly anxiety and depressive disorders. In fact, approximately one-third of patients with Takotsubo cardiomyopathy have a history of psychiatric illness (most often mood or anxiety disorders) prior to their cardiac event [19]. Large-scale cohort studies have reinforced this connection: for example, data from the International Takotsubo Registry and other population analyses show that patients with TTC exhibit significantly higher rates of depression and anxiety compared to matched controls with ordinary coronary heart disease. This analysis of over 24,000 cases found markedly greater prevalence of both anxiety and mood disorders in TTC patients than in those with acute coronary syndrome [20]. Similarly, case–control studies using structured psychiatric assessments have demonstrated that mood disorders, particularly major depressive disorder, are significantly more prevalent among patients with Takotsubo syndrome than among matched controls, while anxiety disorders are also frequently reported, although findings have been less consistent across studies [21]. These observations suggest a strong mind–heart interplay in Takotsubo syndrome, wherein psychological distress and cardiac vulnerability are intertwined.

Mechanistically, acute mental stress or psychiatric episodes are thought to trigger TTC through neurohumoral pathways along the brain–heart axis [22]. A surge in sympathetic nervous system activity and catecholamine release can induce myocardial stunning, which is believed to be the same pathway by which extreme emotions precipitate this cardiomyopathy. Indeed, there are documented instances in which an acute exacerbation of a psychiatric condition (such as a panic attack, severe depressive episode, manic psychosis, or other intense emotional state) immediately preceded and likely precipitated a Takotsubo event. In one case series, patients with TTC were seven times more likely to have a history of anxiety or depression, and the authors observed cases where severe depression, anxiety/agitation from psychosis, or mania appeared to trigger the syndrome [19]. Such findings carry important clinical implications: they highlight the need for cardiologists and mental health professionals to be aware of the association between Takotsubo cardiomyopathy and psychiatric factors, to screen patients for underlying anxiety or depression, and to consider stress management as part of preventive and therapeutic strategies.

### 1.3. Gaps in the Literature and Rationale for the Review

Despite the recognized association between psychological factors and Takotsubo cardiomyopathy, significant gaps remain in our understanding of these comorbidities. Most studies to date linking anxiety or depression with TTC have been retrospective in design or based on small case series. This limits the strength of the evidence and leaves several key questions unresolved. One major area of uncertainty is the relative contribution of anxiety versus depressive disorders to Takotsubo risk. Some smaller investigations have suggested that pre-existing anxiety disorders (but not mood disorders) are uniquely associated with developing Takotsubo syndrome, whereas others have found the opposite or that both types of disorders are implicated. Larger registry studies tend to indicate that both anxiety and depression are over-represented in TTC populations, but the inconsistency in smaller studies highlights a need for clarification. Moreover, the mechanisms connecting chronic anxiety or depression to the acute stress cardiomyopathy remain poorly elucidated. While the catecholamine surge hypothesis offers a plausible explanation, the precise neurocardiological pathways (involving central autonomic regulation, the limbic system, etc.) are not fully mapped out and may involve predispositions that have yet to be identified. Another gap in the literature is how these psychiatric comorbidities impact patient outcomes and management after a Takotsubo event. It has been observed that having a psychiatric history might increase the risk of Takotsubo recurrence in some patients, but it is not clear whether long-term mortality or recovery differ in this group, and data on prognostic implications are limited. There are currently no specific guidelines on managing Takotsubo patients with co-occurring anxiety or depression, and no consensus on whether targeted psychiatric or psychosocial interventions could improve cardiac outcomes. Overall, the existing literature is limited and sometimes underpowered, underscoring the need for more robust research on the heart–mind relationship in Takotsubo syndrome. In light of these gaps, the present scoping review is undertaken to systematically map the available evidence on the comorbidities between anxiety, depression, and Takotsubo cardiomyopathy. By charting what is known and identifying the limitations in current knowledge, this review aims to help bridge the minds and hearts to help informing clinicians and researchers where further investigation is needed to fully understand and address this intriguing cardio-psychiatric connection.

### 1.4. Objectives and Hypotheses

The primary objective of this scoping review is to systematically map the current body of evidence regarding the comorbidity between TTC and psychiatric disorders, with a specific focus on anxiety and depressive disorders. This review aims to synthesize clinical, epidemiological, and psychometric findings from human studies that investigate the prevalence, nature, and implications of these psychiatric comorbidities in TTC populations. By analyzing diverse study designs, the review seeks to provide a comprehensive overview of how emotional and affective disturbances interact with this stress-induced cardiomyopathy. In light of the emerging literature suggesting that psychiatric vulnerability may not only coexist with but also precipitate Takotsubo episodes, we hypothesize that anxiety and depressive disorders are significantly overrepresented in TTC populations compared to other cardiac cohorts (e.g., acute coronary syndrome). Furthermore, we posit that methodological heterogeneity, underpowered studies, and underutilization of standardized psychiatric assessments have contributed to fragmented findings in the field. Through this review, we aim to (1) identify trends in psychiatric comorbidities reported in TTC studies, (2) evaluate the use and consistency of psychometric instruments, (3) examine the temporal and causal relationships proposed between psychiatric symptoms and TTC onset, and (4) outline gaps in the evidence base to guide future interdisciplinary research. Finally, the goal is to inform both cardiological and psychiatric practice by highlighting the importance of screening for affective disorders in patients presenting with Takotsubo cardiomyopathy and to pave the way for integrated, biopsychosocial approaches to management and prevention.

## 2. Materials and Methods

### 2.1. Search Strategies

To comprehensively map the scientific literature addressing the intersection of TTC and psychiatric comorbidities (specifically anxiety and depressive disorders), a structured scoping review was conducted. The search included studies published up to December 2025 and was executed across four major electronic databases: MEDLINE (via PubMed), Embase and Web of Science. Google Scholar was also included to find relevant studies. The review adhered to the PRISMA-ScR (Preferred Reporting Items for Systematic Reviews and Meta-Analyses extension for Scoping Reviews) guidelines [23]. The study was not preregistered.

The search strategy was developed through a systematic, iterative process incorporating both natural language terms and controlled vocabulary (e.g., MeSH and Emtree headings) to ensure sensitivity and breadth. The strategy included cardiac terms such as “Takotsubo cardiomyopathy,” “stress cardiomyopathy,” “apical ballooning syndrome,” and “broken heart syndrome,” in combination with psychiatric descriptors including “anxiety,” “depression,” “affective disorders,” “mood disorders,” and “psychiatric comorbidity.” Boolean operators were used to combine concept clusters, and truncation techniques were applied where appropriate to broaden search capture. The objective was to retrieve studies addressing either pre-existing psychiatric diagnoses in Takotsubo patients, psychiatric symptoms as triggers for Takotsubo events, or psychological outcomes following a Takotsubo diagnosis. Search strategies were initially piloted on MEDLINE and revised based on preliminary yield and relevance. All searches were conducted by the principal reviewer (AH), with oversight and duplicate verification by a secondary reviewer to ensure methodological consistency and reproducibility. Complete search strings for each database, and a PRISMA-ScR checklist detailing methodological compliance is provided in Supplementary Materials.

### 2.2. Study Eligibility

Studies were included in this review if they met the following eligibility criteria: (1) the study population comprised individuals diagnosed with an anxiety disorder and/or a depressive disorder; (2) participants had experienced at least one confirmed episode of TTC; (3) the co-occurrence of psychiatric and cardiac diagnoses did not have to be simultaneous (lifetime comorbidities were accepted); and (4) the temporal sequence of diagnoses (i.e., whether psychiatric symptoms preceded or followed TTC) was not a criterion for inclusion. A diagnosis of TTC was considered confirmed when the original study reported a clinician-established diagnosis based on accepted diagnostic criteria (e.g., Mayo Clinic or InterTAK criteria) or equivalent contemporary diagnostic standards. Studies were excluded if they discussed anxiety, depression, or Takotsubo cardiomyopathy in isolation without explicitly examining their comorbid or intersecting relationships. Additionally, unpublished manuscripts, dissertations, or other forms of gray literature were excluded from consideration. To ensure linguistic consistency and minimize misinterpretation, only studies published in English or French were retained for full-text review. Studies in other languages were excluded to reduce the risk of translation-related bias.

### 2.3. Data Extraction

A structured data extraction process was undertaken using a standardized coding grid developed in Microsoft Excel to ensure methodological replicability. This framework was specifically designed to the objectives of the review and allowed for consistent collection of relevant variables across all included studies. Data extraction was conducted by two independent reviewers (MTN and MF), who systematically reviewed each eligible article and recorded pertinent information into the grid. Discrepancies or uncertainties encountered during the extraction process were resolved through discussion between the two reviewers, with additional input from a third team member (AH) when consensus could not be immediately achieved.

The extraction grid was designed to capture a broad range of study-level variables. Bibliographic information included author names, publication year, country of origin, and study type (e.g., case series, comparative cohort). Population-level descriptors encompassed psychiatric and cardiologic characteristics such as the presence of anxiety or depressive disorders, diagnosis of Takotsubo cardiomyopathy, sample size, participant age range or mean age, and gender distribution. Additional variables recorded included the nature of psychiatric comorbidities (e.g., diagnostic categories or syndromes), tools or scales used for psychological assessment, and the main outcomes reported in relation to the interplay between psychological and cardiac dimensions. Furthermore, data were extracted on study limitations, either as explicitly acknowledged by the original authors or inferred by the reviewers, in order to facilitate later appraisal of evidence quality and generalizability.

### 2.4. Quality Assessment

To evaluate the methodological quality of the included studies, a formal quality appraisal was conducted using the Joanna Briggs Institute (JBI) critical appraisal tools, selected according to the specific design of each study [24]. Two reviewers (MTN and AH) carried out this appraisal independently, applying the appropriate JBI instrument for each study type, including observational cohorts, cross-sectional investigations, case series, and mixed-methods designs. These tools offer structured criteria to assess the internal validity and transparency of studies, with domains focused on participant selection, exposure and outcome measurement, handling of confounding variables, and the adequacy of statistical or analytical methods. In cases where discrepancies arose in the assessment scores, reviewers discussed the points of divergence and reached agreement through collaborative deliberation. For studies employing mixed-methods approaches, the qualitative and quantitative components were appraised individually using their respective JBI checklists to ensure comprehensive evaluation. Although no studies were excluded solely on the basis of their quality scores, the appraisal results were used to contextualize the strength of evidence and highlight areas where methodological limitations may affect interpretability. A summary of the quality assessment findings is provided in Supplementary Materials. A narrative synthesis highlighting recurrent strengths and weaknesses is provided as part of the results.

## 3. Results

### 3.1. Description of Studies

**Figure 1:**
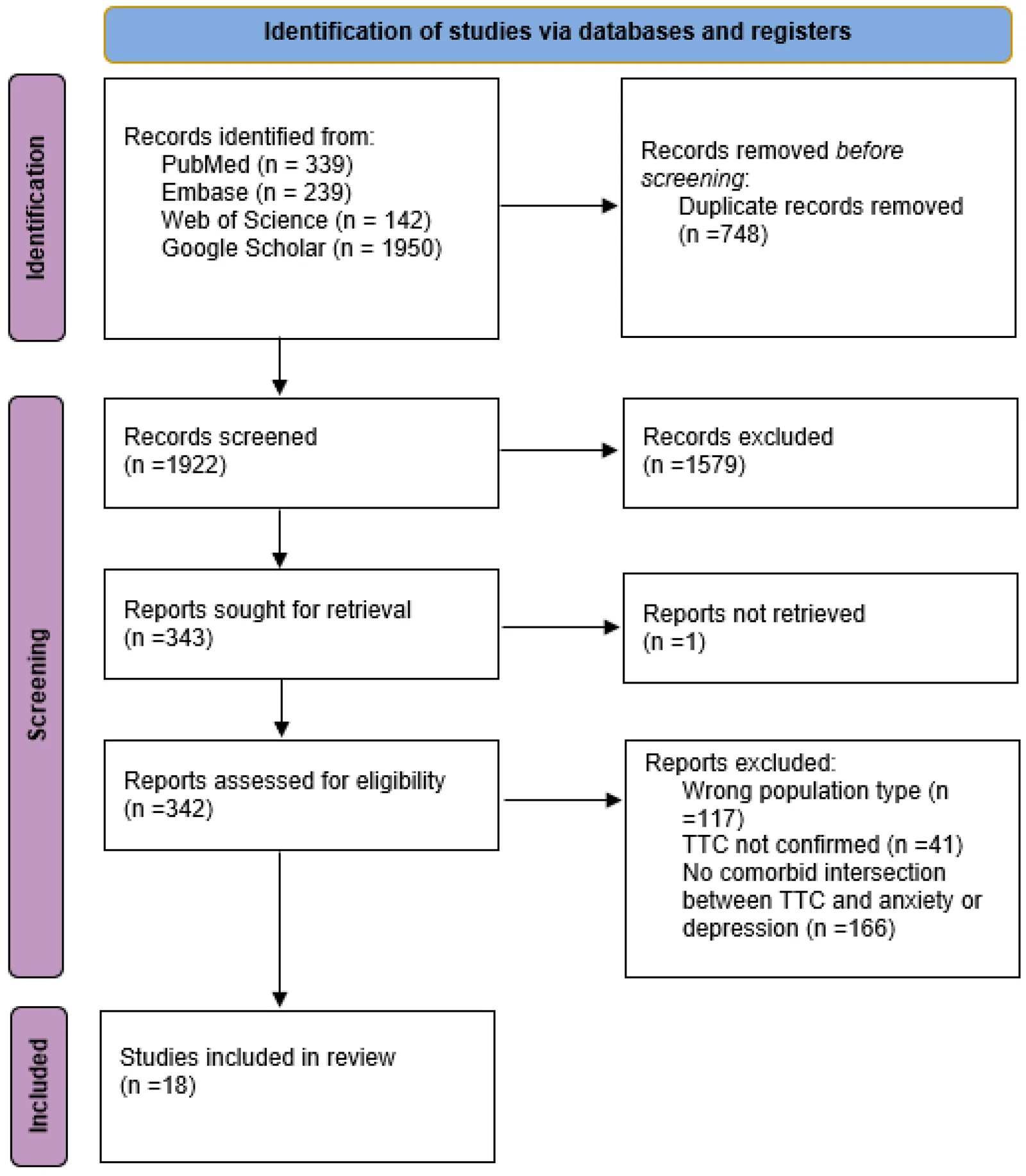
PRISMA (Preferred Reporting Items for Systematic Reviews and Meta-Analyses) flowchart for the inclusion of studies.

A total of 2670 records were retrieved through systematic searches of Embase, Web of Science, MEDLINE, and Google Scholar. After removing 748 duplicate entries, 1922 unique records were screened for eligibility. During the initial screening phase based on titles and abstracts, 1579 studies were excluded for failing to meet basic inclusion criteria. Full texts of 342 potentially relevant articles were retrieved and assessed in detail; one report could not be accessed despite repeated attempts. Of the 341 reports reviewed, 117 were excluded due to involving non-relevant populations, 41 lacked clear diagnostic confirmation of TTC, and 166 did not investigate the comorbid relationship between TTC and anxiety or depression. Following this selection process, 18 studies met all predefined criteria and were included in the final scoping review. A comprehensive breakdown of the study selection process is illustrated in Figure 1, and the detailed characteristics of included studies are presented in Table 1.

### 3.2. Main Outcomes

This scoping review identified 18 peer-reviewed studies examining the comorbidities between TTC and psychiatric conditions, specifically anxiety and depressive disorders. The majority of included studies were case reports or small observational cohorts, with only a few employing structured psychometric tools to assess psychiatric status. Despite methodological heterogeneity, a consistent pattern emerged across the literature: a significant proportion of TTC patients had either a prior psychiatric diagnosis or active symptoms of anxiety and/or depression at the time of their cardiac event. The timing and nature of psychiatric comorbidities varied, with some studies identifying psychiatric conditions as predisposing risk factors, while others reported post-event psychiatric sequelae. Most frequently reported psychiatric diagnoses included major depressive disorder, generalized anxiety disorder, panic disorder, and adjustment disorder. In several cases, psychotropic medications and external emotional stressors were implicated as potential triggers. Despite growing recognition of the psychiatric–cardiac interplay in TTC, validated psychiatric assessments were underutilized, and psychiatric follow-up was inconsistently reported.

### 3.3. Psychiatric Comorbidity in TTC Populations

Psychiatric comorbidities were highly prevalent across the 18 included studies. Depression was explicitly mentioned in 11 studies while anxiety disorders were noted in 9 [19,25,26,27,28,29,30,31,32,33]. Several reports detailed more severe psychiatric profiles such as dementia with psychosis, panic disorder, and suicidality [30,31,32]. Grief and bereavement were commonly cited precipitants [27,32]. Christensen et al. (2016) identified an overlap between TTC and high neuroticism traits, indicating underlying psychological vulnerability [25]. Moreover, Khera et al. (2016) found significantly higher rates of mood and anxiety disorders in TTC cohorts compared to STEMI patients using a large retrospective sample of over 61,000 individuals [29]. These findings reinforce a consistent association between TTC and pre-existing or concurrent affective illness, with emotional stress or psychiatric instability frequently cited as triggers.

### 3.4. Diagnostic Tools and Measurement of Psychological Symptoms

Despite the centrality of psychiatric comorbidity in TTC, only a subset of studies employed validated instruments to assess psychological states. Christensen et al. (2016) used multiple standardized scales including the WHO-5 Well-Being Index, the Major Depression Inventory (MDI), the Symptoms Checklist-90 (SCL-90), and the Anxiety Symptom Scale (ASS) to quantify depressive and anxiety symptoms, neuroticism, and general psychopathology [25]. TTC patients scored significantly higher than controls, suggesting clinically meaningful psychological distress. Goh et al. (2016) used the Hospital Anxiety and Depression Scale (HADS), finding mean anxiety scores of 6.7 ± 4.7 and depression scores of 6.5 ± 4.1 in TTC patients, indicating borderline to moderate distress levels [28]. However, most studies relied on clinical impressions or retrospective chart review to identify psychiatric conditions, limiting the reliability and comparability of diagnostic data. The absence of systematic screening approaches in the majority of studies hindered the ability to determine prevalence rates or assess the severity of comorbid mental illness consistently across populations.

### 3.5. Temporal and Causal Relationships

The temporal relationship between psychiatric disorders and TTC varied widely among studies, contributing to interpretative complexity. In several cases, affective disorders were longstanding and predated the cardiac event. For example, Christensen et al. (2016) identified lifetime depressive traits in a large cohort, and Corrigan et al. (2011) described individuals with chronic psychiatric histories (e.g., psychotic dementia, schizoaffective disorder) [19,25]. In contrast, Maldonado et al. (2013) reported patients with acute psychiatric crises, such as recent suicidal ideation or panic attacks, which appeared temporally proximate to TTC onset [31]. Muto et al. (2024) described a patient who developed TTC shortly after electroconvulsive therapy (ECT) initiation, raising questions about treatment-related triggers [32]. Konstantinos et al. (2017) and Nayeri et al. (2017) provided evidence that emotional stress (e.g., bereavement) in the absence of a formal diagnosis could still serve as a physiological trigger [27,33]. Only one study explicitly assessed psychiatric symptoms both pre- and post-TTC, and none employed prospective designs. As such, while a pattern of psychological precursors is evident, causality cannot be definitively established, and post-TTC psychopathology remains poorly characterized.

### 3.6. Risk Factors, Psychotropics, and Precipitating Stressors

Acute emotional stressors were frequently described as immediate precipitants of TTC. Muto et al. (2024) reported TTC following a significant personal loss and psychiatric hospitalization, while Khera et al. (2016) and Madias (2016) discussed episodes triggered by emotional distress or psychiatric treatment interventions [29,30,32]. Goh et al. (2016) noted that female TTC patients were more likely to report recent anxiety-provoking events than controls [28]. A few studies hinted at the potential role of psychotropic medications. As an example, Maldonado et al. (2013) and Muto et al. (2024) described patients receiving antidepressants, ECT, or antipsychotics shortly before onset, suggesting possible pharmacological influences [31,32]. Nonetheless, no study systematically evaluated medication class, dosage, or interaction with autonomic function. Reviews identified reported chronic psychological burden, such as cumulative trauma or unresolved anxiety, was rarely assessed beyond anecdotal narratives [34,40]. Despite theoretical links to sympathetic hyperactivation and HPA axis dysfunction, biological pathways underlying TTC’s psychiatric triggers were generally inferred rather than empirically tested [35].

Across the included studies, psychiatric disorders and emotional stress emerged as important features of TTC In the International Takotsubo Registry, psychiatric or neurologic disorders were present in 55.8% of patients with TTC compared with 25.7% of patients with acute coronary syndrome, while physical triggers were more common than emotional triggers and were associated with worse in-hospital outcomes [38]. Similarly, Sobue et al. found that patients with physically triggered TTC had significantly higher in-hospital mortality than those with emotional or no identifiable triggers, with male sex and physical triggers independently predicting mortality [37]. Salmoirago-Blotcher et al. reported that women with TTC had a higher prevalence of pre-existing anxiety disorders than myocardial infarction or healthy controls and experienced greater post-discharge psychological distress and post-traumatic stress symptoms, whereas mood disorders were not significantly different between groups [36]. Finally, Yaqub et al. described a rare case of postpartum depression associated with reversible TTC, suggesting that postpartum hormonal changes and emotional stress may contribute to disease onset in susceptible individuals [39].

### 3.7. Psychotropic Medications, Adverse Effects, and Psychiatric Triggers

Across the included studies, three recurring themes emerged. Psychotropic medications were occasionally implicated in Takotsubo cardiomyopathy, primarily through isolated case reports involving antidepressants (e.g., serotonin–norepinephrine reuptake inhibitors) or other psychiatric treatments, although causal relationships could not be established. Also, adverse psychiatric or treatment-related events were infrequently reported and were largely limited to individual cases, such as Takotsubo syndrome occurring in the context of electroconvulsive therapy or severe psychiatric decompensation. Furthermore, psychiatric triggers were consistently identified across studies, with acute emotional stress, panic attacks, severe depressive episodes, manic episodes, psychosis, and bereavement representing the most frequently reported precipitating factors. Finally, the available evidence suggests that acute psychological stressors are more consistently associated with Takotsubo syndrome than psychotropic medication exposure itself.

### 3.8. Limitations in the Existing Literature

Methodological constraints were common across the dataset. Of the 18 studies, 6 were case reports, and only 4 utilized validated psychiatric assessment tools. Most studies had small sample sizes or lacked matched control groups, limiting generalizability. Several explicitly acknowledged recall bias and selection bias due to recruitment from psychiatric units. Longitudinal outcomes were reported in only a few cases, and follow-up psychiatric care was rarely discussed. Additionally, inconsistencies in psychiatric terminology (e.g., “mood disorder” vs. “depression”) and poor documentation of diagnostic procedures introduced classification challenges. Importantly, none of the studies incorporated integrated care models or recommended formal psychiatric co-management, revealing a substantial gap in collaborative TTC treatment strategies.

### 3.9. Quality Appraisal

Most studies demonstrated moderate to high risk of bias due to small sample sizes, retrospective designs, lack of control groups, and inconsistent use of validated psychiatric measures. For instance, while studies such as Christensen et al. (2016) and Goh et al. (2016) met many quality criteria (including the use of standardized psychiatric tools and clear reporting of methods), others, particularly descriptive case reports, provided limited detail on diagnostic procedures or sampling rationale [25,28]. Importantly, no study met all criteria for methodological transparency, and very few incorporated longitudinal data or systematic psychiatric follow-up.

## 4. Discussion

This scoping review systematically mapped the existing evidence on the comorbidities between anxiety, depression, and TTC across 18 peer-reviewed studies. Using a structured extraction grid and design-specific quality appraisal tools from the Joanna Briggs Institute, we explored how psychiatric disorders intersect with the clinical presentation, diagnosis, and trajectory of TTC. The findings reveal a consistent association between TTC and affective psychopathology (particularly major depressive disorder and anxiety disorders) although the nature and timing of this relationship vary. Approximately two-thirds of studies identified depression, and half reported anxiety as comorbid or prodromal to TTC episodes. Acute emotional stressors such as bereavement, conflict, or panic attacks frequently preceded cardiac decompensation. However, the literature is limited by methodological heterogeneity, inconsistent use of validated psychiatric instruments, and underreporting of longitudinal psychiatric outcomes.

Large administrative registry studies (e.g., El-Sayed et al., n = 24,701; Khera et al., n = 53,947) consistently demonstrated that psychiatric disorders are substantially more prevalent among patients with Takotsubo cardiomyopathy than in comparator populations, providing robust epidemiological support for this association. However, these studies relied primarily on ICD coding, lacked standardized psychiatric assessments, and provided limited information regarding symptom severity, temporality, or causal mechanisms. Medium-sized clinical cohorts (e.g., Nayeri et al., n = 306; Christensen et al., n = 173; Goh et al., n = 184; Konstantinos et al., n = 84; Pozzi et al., n = 50; Salmoirago-Blotcher et al., n = 107) offered more detailed clinical and psychiatric characterization, often using validated instruments such as the Hospital Anxiety and Depression Scale (HADS), Mini International Neuropsychiatric Interview (MINI), or other structured assessments. These studies provided stronger evidence regarding the types of psychiatric disorders associated with Takotsubo cardiomyopathy but remained limited by retrospective designs, modest sample sizes, and potential selection bias. Finally, case reports and case series (e.g., Corrigan et al., Maldonado et al., Madias, and Muto et al.) illustrated uncommon but clinically informative scenarios, including TTC occurring during severe depressive episodes, mania, psychosis, electroconvulsive therapy, or following antidepressant exposure. Although these observations cannot establish causality, they generate clinically relevant hypotheses regarding potential psychiatric triggers and treatment-related precipitants that warrant prospective investigation.

These findings corroborate existing epidemiological research demonstrating elevated rates of psychiatric comorbidities in TTC populations. For instance, Templin et al. (2015) analyzed data from the International Takotsubo Registry and reported that over 40% of TTC patients had a history of psychiatric illness, most often mood and anxiety disorders [38]. Similarly, Summers et al. (2010) found that emotional stressors were twice as common in TTC patients compared to those with acute myocardial infarction [41]. The results of our review build on these observations by emphasizing that comorbidity is not merely coincidental but may reflect shared neurobiological mechanisms, including autonomic dysregulation, catecholaminergic surges, and HPA axis activation. This reinforces the concept of TTC as a psychocardiological condition wherein affective dysregulation and cardiac vulnerability intersect.

Moreover, the variable timing of psychiatric symptomatology (whether predisposing, concurrent, or subsequent to the cardiac event) aligns with recent biopsychosocial frameworks that consider TTC as a dynamic disorder influenced by both internal and external stress–response pathways. For example, Wittstein et al. (2005) demonstrated elevated plasma catecholamine levels in TTC patients compared to STEMI controls [42], implicating emotional distress as a potential precipitant. Our review extends this by noting that even in the absence of formal psychiatric diagnoses, many TTC patients were exposed to significant psychological stressors. This supports the relevance of subclinical affective distress and personality traits (e.g., neuroticism) as potential risk amplifiers. These insights underscore the importance of routine psychosocial screening in TTC management protocols.

Despite these observations, psychiatric evaluation and follow-up remain inconsistently implemented in clinical practice. Most studies in this review did not incorporate psychiatric referral or structured mental health interventions post-TTC, and none evaluated the impact of psychopharmacological or psychotherapeutic strategies on recovery or recurrence. This lack of integrated care contrasts with calls in the recent literature for cardiopsychological models of treatment. For example, Mäenpää’s study. advocate for post-discharge psychological support in TTC patients to mitigate anxiety, prevent recurrence, and improve health-related quality of life [43]. Given the reversible yet serious nature of TTC, a more proactive psychiatric component could meaningfully enhance outcomes, particularly in high-risk or recurrent cases.

While routine mental health assessment may appear challenging in busy cardiology settings, several validated ultra-brief screening instruments are specifically designed for rapid clinical use. The Patient Health Questionnaire-2 (PHQ-2) and the Generalized Anxiety Disorder-2 (GAD-2), each consisting of only two questions and requiring less than one minute to complete, have demonstrated good sensitivity for identifying patients who may require more comprehensive psychological evaluation. In patients with Takotsubo cardiomyopathy, these instruments could be incorporated into the initial hospitalization, outpatient follow-up visits, or cardiac rehabilitation programs without substantially increasing clinical workload. Positive screens should not be considered diagnostic but rather as indicators for further assessment using more comprehensive instruments (e.g., PHQ-9 or GAD-7) or referral to primary care, psychology, or psychiatry services when clinically appropriate. Such a stepped-care approach may facilitate earlier recognition of psychiatric comorbidities while remaining feasible within routine cardiology practice.

This review is not without limitations. Although a comprehensive database search was conducted, our exclusion of the gray literature and non-English or French publications may have omitted relevant findings. Restricting the review to English- and French-language publications may have introduced language bias, particularly given that Takotsubo cardiomyopathy was first described in Japan, where relevant studies published exclusively in Japanese may have been missed. Furthermore, the heterogeneity of study designs, small sample sizes, and reliance on retrospective or descriptive methods limited the generalizability of conclusions. Also, the inconsistent use of validated psychiatric tools across studies restricted our ability to quantify prevalence rates or perform meta-synthesis. Additionally, while we applied the JBI quality criteria, the scoping nature of the review precluded formal risk-of-bias weighting or inferential conclusions. Nonetheless, the review highlights important knowledge gaps and underscores the need for interdisciplinary, longitudinal research to better characterize and manage psychiatric comorbidities in Takotsubo cardiomyopathy.

## 5. Conclusions

This scoping review highlights a consistent and clinically meaningful association between TTC and psychiatric disorders, particularly anxiety and depression. Across 18 studies, affective disturbances were frequently reported either as antecedents, triggers, or sequelae of TTC episodes. Despite this, psychiatric assessment remains inconsistently integrated into diagnostic and treatment protocols, and only a minority of studies employed validated psychometric tools. Emotional stress, whether from bereavement, interpersonal conflict, or pre-existing psychiatric illness, emerged as a recurrent precipitant, supporting the theory of neurocardiogenic susceptibility mediated via the brain–heart axis. However, heterogeneity in study design, the predominance of retrospective data, and lack of longitudinal follow-up restrict our understanding of causality, prognosis, and intervention efficacy. The findings underscore the need for routine mental health screening in TTC patients and support the development of integrated cardiopsychological care models. Future research should prioritize prospective, interdisciplinary studies that clarify temporal relationships, identify modifiable psychological risk factors, and evaluate the impact of psychiatric treatments on cardiac outcomes in TTC populations.

## Figures and Tables

**Table 1:** Details of the included studies.

Authors	Population	Article Type	Psychiatric Comorbidity	Scales Used	Main Outcomes	Limitations (as per the Authors of the Study)	Risk of Bias	Overall Quality Assessment
Christensen et al., 2016 [25]	Women with Takotsubo 64–77 N = 173 Copenhagen	Comparative study	Anxiety Depression Neuroticism	WHO-5 ENS MDI ASS SCL-90	TTC patients reported higher levels of anxiety than STEMI patients (mean score of ASS was 50% higher in TTC group *p* = 0.007). TTC patients reported comparable levels of well-being, neuroticism and depression than STEMI patients. TTC patients improved psychologically during the post-recovery phase when compared to TTC patients (correlation Time and WHO-5 r = 0.28 for TTC group vs. correlation Time and WHO-5 r = −0.03 for STEMI group).	Small sample size. Self selection bias. Impossibility to know the direction of causality of the psychopathology–TTC association.	Moderate risk. JBI case–control/cross-sectional domains: clear group definition and validated psychometric scales, but self-selection, small sample, and temporality cannot be established.	Moderate
Corrigan et al., 2011 [19]	Women with Takotsubo 53–67 N = 4 USA	Case series study	Dementia with psychotic disorder Adjustment disorder Major depression Bipolar affective disorder	None	Exacerbation of underlying psychiatric illness can lead to TTC. (no objective data presented)	Small sample size. Lack of control group.	High risk. JBI case series domains: very small n = 4, no control group, no standardized psychiatric assessment or objective scales, and limited ability to establish causal attribution.	Low
El-Sayed et al., 2012 [26]	Patients with Takotsubo N = 24,701 USA	Comparative study	Anxiety Mood disorder Delirium/dementia	None	Patients with TTC were more likely to be White, female gender and have higher incomes than those with myocardial infarction or orthopedic patients (*p* < 0.0001 for the three factors). Patients with SC were less likely to have cardiovascular risk factors compared to MI and orthopedic controls but were more likely to have other pathologic histories. Those pathologic histories are cerebrovascular accidents, drug abuse, anxiety disorders, mood disorders, malignancy, chronic liver disease, and sepsis.	Possible improper classification of patients with TTC using ICD-9-CM diagnostic criteria. Improper physician diagnoses of the syndrome. Selection bias. Impossible to know the direction of causality of comorbidities–TTC.	Moderate risk. JBI analytical cross-sectional/administrative database domains: very large sample and comparator group, but exposure/outcome identification relied on ICD-9-CM codes with possible misclassification and residual confounding.	Moderate
Konstantinos et al., 2017 [27]	Patients initially presenting with Takotsubo N = 84 Germany	Comparative cohort study	Anxiety Grief	None	Significantly lower event-free survival rate over a mean follow-up of 5 years in the physical stress group than in the emotional stress group (log-rank, *p* < 0.05).	The main outcome statement is not necessarily a proof that emotional stress is linked with lower in-hospital events. It may possibly just be an evidence that physical stress is a condition that increases in-hospital events.	Moderate to high risk. JBI cohort domains: clinically relevant cohort and follow-up, but unclear confounder control, unclear exposure measurement for anxiety/grief, and limited reporting of attrition and prognostic model details.	Low
Goh et al., 2016 [28]	Patients with cardiovascular events (Takotsubo and ACS) N = 184 USA	Comparative study	Anxiety Depression	HADS	The mean anxiety score was 6.7 ± 4.7 for TTC cases versus 5.4 ± 3.4 for ACS controls (*p* = 0.06). The mean depression score was 4.3 ± 3.7 for TSCM cases versus 4.0 ± 3.1 for controls (*p* = 0.61).	Small sample size. Case–control design. Recall bias, HADS related anxiety/depression secondary to memories reevoked. High prevalence of anxiety/depression in people with ACS. No data on preceding emotional/physical stress in the ACS controls.	Moderate risk. JBI case–control domains: appropriate ACS comparator and HADS scale, but small sample, recall bias, and cross-sectional assessment limit temporality and causal inference.	Moderate
Khera et al., 2016 [29]	Patients with diagnosis of Takotsubo N = 53,947 USA	Cross-sectional descriptive study	Anxiety Mood disorder	None	There is more than a three-fold increase in hospitalization for TTC in the United States over a 6 year period, 2007 through 2012. This fact likely represents an increasing recognition of this disease. Relevant secondary outcome to this study: 29.7% of patients with primary TTC have a psychiatric diagnosis while 26.6% of patients with secondary TTC have a psychiatric diagnosis. (*p* = 0.0007)	Using ICD-9 discharge codes, the presentation details of the patients are unavailable so it is limiting the ability to independently confirm the diagnosis. The NIS data does not provide information on important clinical predictors of outcomes including disease severity, left ventricular ejection fraction and baseline functional status of patients, which can potentially confound the adjustment for patient characteristics. Given the administrative nature of the database, it is not possible to reliably differentiate comorbidities from complications of hospitalization. The NIS does not provide information that can be used to reliably predict pre-hospital events that may have served as likely precipitants.	Moderate risk. JBI prevalence/cross-sectional domains: very large national dataset, but reliance on discharge codes, inability to verify diagnostic criteria, and limited clinical covariate adjustment.	Moderate
Madias 2016 [30]	Takotsubo in patients using normal dosing of ISRN N = 1 USA	Case report	Depression	None	These findings support the theory of apical ballooning caused by catecholamine-induced myocardial stunning. The patient had elevated serotonin/norepinephrine levels secondary to reuptake inhibition leading to TTC.	None declared. Personal analysis and Madias commentary: Small sample. No blood-borne catecholamines measurements made. No further EKGs were made to confirm the well-known effects of venlafaxine on the QRS complex.	High risk. JBI case report domains: single patient, limited baseline information, no rechallenge, no standardized causality assessment, and uncertain competing explanations.	Low
Maldonado et al., 2013 [31]	Patients with Takotsubo having mania N = 1 USA	Case report	Bipolar I disorder	None	In the case of Ms. A, emotional stress may have been triggered by either an acute exacerbation of her underlying bipolar disorder, one of several acute psychosocial stressors, or a combination thereof.	Difficult to know if the acute manic episode was triggered by the discontinuation of lithium therapy, the acute psychosocial stressors, or a combination of both. Small sample. No dosage of catecholamines to prove correlation.	High risk. JBI case report domains: single case with plausible but non-specific temporal association; important competing explanations include medication discontinuation, acute mania, stress, and medical vulnerability.	Low
Muto et al., 2024 [32]	Schizophrenic patients undergoing ECT N = 1 Japan	Case report	Schizophrenia	None	The patient had numerous cases of aspiration pneumonia and malnutrition before ECT was performed, which might have made this case fatal. In conclusion, appropriate supplementation of nutrition and reduction of physical stressors are important to avoid death from TTC caused by ECT.	No tests that suggest the possibility of developing TTC was performed directly after ECT to suggest that the desaturation of the patient was caused by TTC.	High risk. JBI case report domains: single patient, limited pre-event cardiac risk assessment, multiple medical confounders, and no systematic method to estimate risk before ECT.	Low
Nayeri et al., 2017 [33]	Diagnosed patients with Takotsubo N = 306 USA	Comparative cohort study	Mood, anxiety, or schizophrenia- spectrum illness.	None	114 (37%) patients had a pre-existing mood, anxiety, or schizophrenia-spectrum illness at the time of index TTC diagnosis, accounting for a total 151 diagnoses. Pre-existing psychiatric illness was not associated with increased 30-day (*p* = 0.320) or long-term (*p* = 0.621) mortality. Pre-existing psychiatric illness was associated with higher risk of recurrent TTC (*p* < 0.001).	Retrospective cohort design = only association and can’t infer causation. Small cohort size. Insufficient statistical power. Potential underdiagnosis or misdiagnosis of psychiatric illness in the cohort.	Moderate risk. JBI cohort domains: consecutive clinical sample and relevant outcome follow-up, but retrospective design, confounding by illness severity and treatment, incomplete psychiatric characterization, and association only.	Moderate
Oliveri et al., 2020 [34]	N/A	Narrative review	Anxiety disorders and depression	N/A	Anxiety disorders, but not depression, are associated with a higher occurrence of TTC. There is a link between anxiety, TTC, and inflammation leading to increased sympathetic activity. The pre-admission prevalence of anxiety disorders is more prevalent in patients who developed TTC than in the healthy population and STEMI/ACS controls. Depression is not associated with a higher occurrence of TTC activity. Patients with pre-admission psychiatric disorders have a higher risk of recurrent TTC and are more predisposed to suffer from acute in-hospital complications.	No review of articles published before 2010. Only reviews abstracts written in English. Many articles reviewed had small sample sizes. No specific clinical trials that deal with psychiatric disorders/anxiety disorders and TTC in literature. Diagnosis of anxiety disorders or depression was not established by using the same diagnostic criteria in each article reviewed.	High/not directly applicable for primary-study RoB. Narrative review; no reproducible comprehensive search or formal critical appraisal, limited language/date scope, and high selection bias.	Low
Pozzi et al., 2022 [35]	50 female patients, 25 with a previous diagnosis of TTC (cases) and 25 patients with a history of ACS (controls) N = 50 Italy?	Retrospective observational case–control study	Mood disorders (Major Depressive Disorder, Bipolar Disorder, Dysthymia), Anxiety disorders (Generalized Anxiety Disorder, Panic Disorder, Agoraphobia), other disorders (PTSD, Eating Disorders, Alcohol Abuse Disorder), substance abuse, previous stressful events, Type D personality.	InterTAK criteria for TTC diagnosis and European guidelines for diagnosis of control group (acute coronary syndrome). Psy: ERQ, CD-RISC, DS-14, PID-5, MINI, DSM-V, SRRS.	The frequency of psychiatric disorders was significantly greater in patients with TTC compared with ACS patients. TTC group showed a higher prevalence of mood disorders (MD), i.e., Major Depressive Disorder, Bipolar Disorder, and Dysthymia (*p* < 0.001); anxiety disorders (AD), i.e., Generalized Anxiety Disorder, Panic Disorder and Agoraphobia (*p* = 0.001); and other disorders, i.e., Post-Traumatic Stress Disorder, Eating disorders and Alcohol Use Disorder (*p* = 0.034), compared with the control group. There was no difference between the groups in previous stressful events (*p* = 0.07) and Type D personality (negative affectivity and social inhibition; *p* = 0.38). After multivariate logistic regression adjusted model, mood disorders were independently associated with TTC, OR 16.9 (95% CI 2.2–127).	Small-size population. Retrospective nature of the study gives an inferior level of evidence. Lack of follow-up data does not allow to determine whether psychiatric morbidity also have a prognostic role.	Moderate to high risk. JBI case–control domains: clear diagnostic criteria and psychiatric evaluation, but small retrospective sample, potential selection bias, and limited adjustment for confounding.	Low
Salmoirago-Blotcher et al., 2016 [36]	107 participants (mostly women, control group were only women). Takotsubo cases (N = 45), 2 control groups: Controls with MI (N = 32) and healthy controls (HC) (N = 30), USA	Case–control study and cross-sectional descriptive study	Mood (depressive disorder, bipolar disorder) and anxiety disorder (for the case–control study) Post-discharge psychological distress (depression, anxiety, stress, PTSD) and personality characteristics (hostility, optimism, Type D (“distressed”) personality) (for the cross-sectional study).	Case–control: Mayo Clinic Criteria for TC diagnosis. The definitions of the different psychiatric conditions were based on DSM-IV-TR criteria. Cross-sectional study: HADS, Perceived stress scale, IES, Cook-Medley Hostility Inventory, Life Orientation Test, DS14 Questionnaire	Case–control: TTC patients were significantly more likely to have a history of anxiety disorder (24.4%) than MI (12.5%) and HC (0.0%, *p* = 0.01). The prevalence of mood disorders was similar across groups (*p* = 0,87). Cross-sectional: Psychological distress remained significantly higher among TTC cases compared to HC (b = 2.44, SE = 1.28, *p* < 0.05) but did not differ between TTC and MI (b = 1.20, SE 1.18; *p* = 0.31). Differences in perceived stress were also no longer significant, indicating that socio-economic status was an important confounder of this association. PTSD symptoms remained significantly higher among TTC women compared to both MI and HC (TTC vs. HC: b = 0.92, SE = 0.34, *p* < 0.01; TC vs. MI: b = 0.55, SE = 0.29, *p* < 0.05). Optimism and hostility scores were similar across groups. As for type personality, TTC and MI women had significantly higher scores for both negative affectivity and social inhibition compared to HC. In adjusted models, differences in negative affectivity were no longer significant, while social inhibition was significantly higher in TTC.	Small sample size. Recall bias. The HC group was recruited from a registry of research volunteers. Only females were enrolled.	Moderate risk. JBI case–control/cross-sectional domains: appropriate diagnostic criteria and comparison groups, but small sample, recall bias, recruitment differences between controls, and limited control for confounders.	Moderate
Sancassiani et al., 2018 [21]	95 patients (19 Takotsubo Syndrome cases and 76 subjects with no diagnosis of Takotsubo Syndrome, Italy.	Case–control study	Major Depressive Disorder (MDD), Bipolar Disorder (BD) (including Bipolar I and Bipolar II), Post-Traumatic Stress Disorder (PTSD), Panic Disorder (PD) and Generalized Anxiety Disorder (GAD).	Mayo Clinic Criteria for Takotsubo diagnosis for cases. Psy: ANTAS, SCID-I, ICD-10.	All psychiatric disorders evaluated in this study showed a higher prevalence in cases, but only in MDD, the difference reached statistical significance (*p* = 0.014) as well as the association of people with at least one mood disorder diagnosis and TTC (*p* = 0.002). Lifetime prevalence of people with at least one anxiety disorder without mood disorder showed a low frequency in patients with TTC. The difference versus controls did not reach statistical significance (*p* = 0.57).	Cases were diagnosed with Takotsubo syndrome in a clinical setting using established diagnostic criteria, whereas control participants were classified as free of TTC based on medical history and previous clinical investigations rather than systematic cardiac evaluation. Consequently, some undiagnosed TTC cases may have been misclassified as controls. However, because TTC is a rare condition, the likelihood of such misclassification is low. Moreover, any non-differential misclassification of controls would be expected to bias the association toward the null, potentially underestimating rather than exaggerating the observed association between TTC and mood disorders.	Moderate to high risk. JBI case–control domains: structured psychiatric assessment, matched controls, and clear TTC criteria, but very small case group, possible diagnostic ascertainment differences, and limited statistical power.	Low
Sobue et al., 2017 [37]	N = 82 patients diagnosed with Takotsubo, Japan	Prospective cohort study	Non-specified psychiatric comorbidities. Emotional triggers to TC such as fear/panic/anxiety, severe argument/frustration, death of relative/pet, interpersonal conflict, workplace problems, malignancy diagnosis, involvement in car accident, divorce.	None for the non-physical triggers	The in-hospital mortality significantly differed between the physical and non-physical trigger types (*p* = 0.007)	Single tertiary care center. Two possible confounding factors.	Moderate risk. JBI cohort domains: prospective design and consecutive TTC patients, but single-center sample, non-standardized assessment of psychiatric/non-physical triggers, and residual confounding.	Moderate
Templin et al., 2015 [38]	N = 1750 from the International Takotsubo Registry, a consortium of 26 centers in Europe and the United States for the 1st part of the study and n = 455 in each group for TC patients and age- and sex-matched patients with an acute coronary syndrome (Zurich Acute Coronary Syndrome Registry) who either fulfilled the third universal definition of myocardial infarction or had unstable angina caused by obstructive coronary artery disease.	Cohort study	Emotional triggers: Grief/Loss, Panic/Fear/Anxiety, Interpersonal conflict, Anger/Frustration, Financial or employment problems. Psychiatric disorders: Affective disorder, anxiety disorder, reaction to severe stress or adjustment disorder, other psychiatric disorders.	Patients were included in the registry on the basis of the Mayo Clinic diagnostic criteria for this condition (TC). Diagnostic and Statistical Manual of Mental Disorders, fourth edition, for the psychiatric diagnoses in patient records	Physical triggers were more common than emotional triggers (36.0% vs. 27.7%), while 7.8% of patients experienced both. Emotional triggers predominated in women, whereas physical triggers were more frequent in men. Compared with patients with acute coronary syndrome, those with TTC had a significantly higher prevalence of neurologic or psychiatric disorders (55.8% vs. 25.7%, *p* < 0.001). Overall, 42.3% had a psychiatric disorder, approximately half of which were affective disorders, and 27.0% had a history of neurologic disease.	None declared	Low to moderate risk. JBI cohort/registry domains: large multicentre registry with standardized TTC criteria and outcome follow-up, but observational design, potential referral/registry bias, and psychiatric triggers/comorbidities not assessed with structured instruments.	High
Yaqub et al., 2009 [39]	N = 1, 32-year-old Female, USA	Case report	Postpartum depression	None.	TTC occurring in a young woman with postpartum depression is rare and may reflect hormonal changes and catecholamine excess.	None declared	High risk. JBI case report domains: single patient, rare presentation, limited ability to separate postpartum depression, emotional stress, and other medical precipitants; no comparator.	Low
Zvonarev et al., 2019 [40]	N/A	Systematic literature review	Anxiety disorders, depressive disorders, mood disorders and Type D personality	None.	Psychiatric disorders were consistently more prevalent among patients with TTC than controls. Sancassiani et al. found that 84.5% of TTC patients reported at least one traumatic life event, with major depressive disorder (*p* = 0.014) and mood disorders (*p* = 0.002) significantly associated with TTC. Other studies reported higher rates of anxiety (39–56% vs. 5–12% in STEMI), depression (48–73% vs. 26–28% in ACS/STEMI), emotional stress triggers (56% vs. 16% in ACS), Type D personality (76% vs. 32–43% in controls), and anxiety traits (60% vs. 52% in STEMI). In a large administrative study, anxiety (8.9% vs. 3.4%) and mood disorders (15.0% vs. 7.2%) were significantly more common in TTC than acute myocardial infarction.	Most included studies had small sample sizes, and the influence of age, race, and ethnicity on the association between TTC and psychiatric disorders was insufficiently explored. Additionally, TTC was diagnosed prospectively in cases but identified from medical history in controls, creating the potential for undiagnosed TTC cases among controls. Although such misclassification is likely minimal given the rarity of TTC, it could have attenuated the observed associations and limited the generalizability of the findings.	High risk. JBI systematic review domains: broad topic and transparent keywords, but search/selection strategy is insufficiently reproducible, no formal RoB appraisal of included studies, heterogeneous designs summarized narratively, and conclusions may overstate causality.	Low

List of acronyms: ACS: Acute Coronary Syndrome; ANTAS: Advanced Neuropsychiatric Tools and Assessment Schedule; ASS: Adversity and Stress Scale; CD-RISC: Connor–Davidson Resilience Scale; DS-14: Measure of negative affectivity and social inhibition, typical features of Type D personality; ECT: Electroconvulsive Therapy; ENS: Existential Nihilism Scale; ERQ: Emotion Regulation Questionnaire; HADS: Hospital Anxiety and Depression Scale; ICD-9-CM: International Classification of Diseases, Ninth Revision, Clinical Modification; MDD: Major Depressive Disorder; MDI: Major Depression Inventory; MI: Myocardial Infarction; MINI: Mini International Neuropsychiatric Interview; NIS: National Inpatient Sample; PID-5: Personality Inventory for DSM-5; SC: Stress Cardiomyopathy; SCID: Structured Clinical Interview for DSM Disorders; SCL-90: Symptom Checklist-90; SRRS: Social Readjustment Rating Scale; TSCM: Takotsubo Stress Cardiomyopathy; TTC: Takotsubo Cardiomyopathy; TTC: Takotsubo Syndrome; WHO-5: World Health Organization-Five Well-Being Index.

## Data Availability

No new data were created or analyzed in this study. Data sharing is not applicable to this article.

## Supplementary Materials

The following supporting information can be downloaded at https://www.mdpi.com/article/10.3390/jcm15166242/s1; Table S1: Search strategies; Table S2: Preferred Reporting Items for Systematic reviews and Meta-Analyses extension for Scoping Reviews (PRISMA-ScR) Checklist.

## Abbreviations

The following abbreviations are used in this manuscript:

Abbreviation	Definition
ACS	Acute Coronary Syndrome
ANTAS	Advanced Neuropsychiatric Tools and Assessment Schedule
ASS	Anxiety Symptom Scale
BDI	Beck Depression Inventory
CD-RISC	Connor–Davidson Resilience Scale
DSM-IV-TR	Diagnostic and Statistical Manual of Mental Disorders, Fourth Edition, Text Revision
DS-14	Type D Scale-14 (Measure of Negative Affectivity and Social Inhibition)
ECT	Electroconvulsive Therapy
ENS	Existential Nihilism Scale
ERQ	Emotion Regulation Questionnaire
GAD	Generalized Anxiety Disorder
HADS	Hospital Anxiety and Depression Scale
HC	Healthy Controls
ICD-9-CM	International Classification of Diseases, Ninth Revision, Clinical Modification
IES-R	Impact of Event Scale–Revised
JBI	Joanna Briggs Institute
MDD	Major Depressive Disorder
MDI	Major Depression Inventory
MI	Myocardial Infarction
MINI	Mini International Neuropsychiatric Interview
NIS	National Inpatient Sample
PHQ-9	Patient Health Questionnaire-9
PID-5	Personality Inventory for DSM-5
PRISMA-ScR	Preferred Reporting Items for Systematic Reviews and Meta-Analyses Extension for Scoping Reviews
PTSD	Post-Traumatic Stress Disorder
SC	Stress Cardiomyopathy
SCID	Structured Clinical Interview for DSM Disorders
SCL-90	Symptom Checklist-90
SRRS	Social Readjustment Rating Scale
STEMI	ST-Segment Elevation Myocardial Infarction
TTC	Takotsubo Cardiomyopathy
TSCM	Takotsubo Stress Cardiomyopathy
TTC	Takotsubo Syndrome
WHO-5	World Health Organization-Five Well-Being Index
