# Supplementary material for: Bridging Minds and Hearts by Exploring the Comorbidities Between Anxiety, Depression, and Takotsubo Cardiomyopathy: A Scoping Review
Source: J Clin Med. 2026 Aug 12;15(16):6242. doi: 10.3390/jcm15166242 (PMC13513078; doi:10.3390/jcm15166242)
Supplement: Supplementary file 1 [file jcm-15-06242-s001.zip › jcm-4443279-supplementary.pdf]

Supplementary Materials

Table S1 . Search strategies

**Data register**	**Search strings**
PubMed, k = 339	(<br>"Takotsubo Cardiomyopathy"[Mesh]<br>OR takotsubo[tiab]<br>OR "takotsubo syndrome"[tiab]<br>OR "takotsubo cardiomyopathy"[tiab]<br>OR "stress cardiomyopathy"[tiab]<br>OR "stress-induced cardiomyopathy"[tiab]<br>OR "stress-induced myocardial dysfunction"[tiab]<br>OR "broken heart syndrome"[tiab]<br>OR "apical ballooning syndrome"[tiab]<br>OR "apical ballooning"[tiab]<br>)<br>AND<br>(<br>"Depressive Disorder"[Mesh]<br>OR "Depression"[Mesh]<br>OR "Anxiety Disorders"[Mesh]<br>OR "Anxiety"[Mesh]<br>OR depression[tiab]<br>OR depressive[tiab]<br>OR anxiety[tiab]<br>OR anxious[tiab]<br>OR "mood disorder*"[tiab]<br>OR "affective disorder*"[tiab]<br>OR psychiatric[tiab]<br>OR psychiatry[tiab]<br>OR psychological[tiab]<br>OR psychopathology[tiab]<br>OR "mental disorder*"[tiab]<br>OR "mental illness*"[tiab]<br>OR "psychiatric comorbid*"[tiab]<br>)<br>AND<br>(<br>Humans[Mesh]<br>)
Embase, k = 239	(<br>'takotsubo cardiomyopathy'/exp<br>OR 'takotsubo syndrome'<br>OR 'takotsubo cardiomyopathy'<br>OR 'stress cardiomyopathy'

	OR 'stress induced cardiomyopathy'<br>OR 'broken heart syndrome'<br>OR 'apical ballooning syndrome'<br>OR 'apical ballooning'<br>)<br>AND<br>(<br>'depression'/exp<br>OR 'depressive disorder'/exp<br>OR 'anxiety'/exp<br>OR 'anxiety disorder'/exp<br>OR 'mood disorder'/exp<br>OR 'psychiatric disorder'/exp<br>OR 'mental disease'/exp<br>OR depress*:ti,ab<br>OR anxi*:ti,ab<br>OR psychiatric:ti,ab<br>OR psychological:ti,ab<br>OR psychopathology:ti,ab<br>OR "affective disorder*":ti,ab<br>OR "psychiatric comorbid*":ti,ab<br>)<br>AND<br>[human]/lim<br>AND<br>(english OR french)/lim
Web of Science, k = 142	TS=(<br>(<br>takotsubo<br>OR "takotsubo syndrome"<br>OR "takotsubo cardiomyopathy"<br>OR "stress cardiomyopathy"<br>OR "stress-induced cardiomyopathy"<br>OR "broken heart syndrome"<br>OR "apical ballooning syndrome"<br>OR "apical ballooning"<br>)<br>AND<br>(<br>depression<br>OR depressive<br>OR anxiety<br>OR anxious<br>OR "mood disorder*"<br>OR "affective disorder*"<br>OR psychiatric<br>OR psychiatry<br>OR psychological

	OR psychopathology<br>OR "mental disorder*"<br>OR "mental illness*"<br>OR "psychiatric comorbid*"<br>)<br>)
Google Scholar, k = 1950	allintitle:<br><br>Takotsubo OR "stress cardiomyopathy";<br><br>depression OR anxiety OR psychiatric<br>"Takotsubo syndrome" depression<br><br>"Takotsubo syndrome" anxiety<br><br>"stress cardiomyopathy" psychiatric<br><br>"broken heart syndrome" depression<br><br>"broken heart syndrome" anxiety

Table S2. Preferred Reporting Items for Systematic reviews and Meta-Analyses extension for Scoping Reviews (PRISMA-ScR) Checklist

SECTION	ITEM	PRISMA-ScR CHECKLIST ITEM	REPORTED ON PAGE
**TITLE**			
**Title**	**1**	**Identify the report as a scoping review.**	**1**
**ABSTRACT**			
**Structured summary**	**2**	**Provide a structured summary that includes (as applicable): background, objectives, eligibility criteria, sources of evidence, charting methods, results, and conclusions that relate to the review questions and objectives.**	**1**
**INTRODUCTION**			

SECTION	ITEM	PRISMA-ScR CHECKLIST ITEM	REPORTED ON PAGE
Rationale	3	Describe the rationale for the review in the context of what is already known. Explain why the review questions/objectives lend themselves to a scoping review approach.	2
Objectives	4	Provide an explicit statement of the questions and objectives being addressed with reference to their key elements (e.g., population or participants, concepts, and context) or other relevant key elements used to conceptualize the review questions and/or objectives.	4
METHODS			
Protocol and registration	5	Indicate whether a review protocol exists; state if and where it can be accessed (e.g., a Web address); and if available, provide registration information, including the registration number.	N/A
Eligibility criteria	6	Specify characteristics of the sources of evidence used as eligibility criteria (e.g., years considered, language, and publication status), and provide a rationale.	5
Information sources*	7	Describe all information sources in the search (e.g., databases with dates of coverage and contact with authors to identify additional sources), as well as the date the most recent search was executed.	5
Search	8	Present the full electronic search strategy for at least 1 database, including any limits used, such that it could be repeated.	5

SECTION	ITEM	PRISMA-ScR CHECKLIST ITEM	REPORTED ON PAGE
Selection of sources of evidence†	9	State the process for selecting sources of evidence (i.e., screening and eligibility) included in the scoping review.	5
Data charting process‡	10	Describe the methods of charting data from the included sources of evidence (e.g., calibrated forms or forms that have been tested by the team before their use, and whether data charting was done independently or in duplicate) and any processes for obtaining and confirming data from investigators.	6
Data items	11	List and define all variables for which data were sought and any assumptions and simplifications made.	6
Critical appraisal of individual sources of evidence§	12	If done, provide a rationale for conducting a critical appraisal of included sources of evidence; describe the methods used and how this information was used in any data synthesis (if appropriate).	6
Synthesis of results	13	Describe the methods of handling and summarizing the data that were charted.	4-6
**RESULTS**			
Selection of sources of evidence	14	Give numbers of sources of evidence screened, assessed for eligibility, and included in the review, with reasons for exclusions at each stage, ideally using a flow diagram.	7

SECTION	ITEM	PRISMA-ScR CHECKLIST ITEM	REPORTED ON PAGE
Characteristics of sources of evidence	15	For each source of evidence, present characteristics for which data were charted and provide the citations.	7
Critical appraisal within sources of evidence	16	If done, present data on critical appraisal of included sources of evidence (see item 12).	7-36
Results of individual sources of evidence	17	For each included source of evidence, present the relevant data that were charted that relate to the review questions and objectives.	7-36
Synthesis of results	18	Summarize and/or present the charting results as they relate to the review questions and objectives.	36
**DISCUSSION**			
Summary of evidence	19	Summarize the main results (including an overview of concepts, themes, and types of evidence available), link to the review questions and objectives, and consider the relevance to key groups.	36
Limitations	20	Discuss the limitations of the scoping review process.	37
Conclusions	21	Provide a general interpretation of the results with respect to the review questions and objectives, as well as potential implications and/or next steps.	38
**FUNDING**			

SECTION	ITEM	PRISMA-ScR CHECKLIST ITEM	REPORTED ON PAGE
Funding	22	Describe sources of funding for the included sources of evidence, as well as sources of funding for the scoping review. Describe the role of the funders of the scoping review.	38

From: Tricco AC, Lillie E, Zarin W, O'Brien KK, Colquhoun H, Levac D, et al. PRISMA Extension for Scoping Reviews (PRISMAScR): Checklist and Explanation. Ann Intern Med. 2018;169:467–473. doi: 10.7326/M18-0850.
